# Tailoring Dialdehyde Bacterial Cellulose Synthesis for Versatile Applications

**DOI:** 10.3390/polym17131836

**Published:** 2025-06-30

**Authors:** Krittanan Kadsanit, Malinee Sriariyanun, Muenduen Phisalaphong, Suchata Kirdponpattara

**Affiliations:** 1 Department of Chemical Engineering, Faculty of Engineering, King Mongkut’s University of Technology North Bangkok, Bangkok 10800, Thailand; krittanankadsanit@gmail.com; 2 The Sirindhorn International Thai-German Graduate School of Engineering, King Mongkut’s University of Technology North Bangkok, Bangkok 10800, Thailand; malinee.s@tggs.kmutnb.ac.th; 3 Biorefinery and Process Automation Engineering Center (BPAEC), King Mongkut’s University of Technology North Bangkok, Bangkok 10800, Thailand; 4 Department of Chemical Engineering, Faculty of Engineering, Chulalongkorn University, Bangkok 10330, Thailand; muenduen.p@chula.ac.th

**Keywords:** bacterial cellulose, degree of oxidation, dialdehyde bacterial cellulose, gelatin, optimization, RSM

## Abstract

Dialdehyde bacterial cellulose (DBC) has been implemented in versatile applications. DBC was prepared from bacterial cellulose (BC) through periodate oxidation with varying parameters, including the mole ratio of BC and NaOI_4_, temperature, and reaction time. The relationship between the degree of oxidation (DO)/aldehyde content and these parameters was proposed as a quadratic equation to predict the oxidation conditions needed to achieve a specific DO using Response Surface Methodology (RSM). The chemical structure and morphology of DBC were influenced by DO. DBC with different DO levels was used as a crosslinker and a reinforcing agent for gelatin sponge fabrication. Results indicated that a high DO of DBC could enhance the tensile strength and structural stability of the gelatin matrix. Selecting the proper DO level could control the morphological structure of the gelatin sponge, which is crucial for biomedical applications.

## 1. Introduction

Bacterial cellulose (BC) or microbial nanocellulose is a polysaccharide with a linear chain of β-1,4 linked d-glucose units. Via metabolic pathway, pure cellulose nanofibrils are extruded through the cell wall of *Acetobacter xylinum* and then packed in the form of a jelly-like pellicle with high crystallinity, a high degree of polymerization, and high mechanical strength [1]. Consequently, BC has been widely used as a material-based composite and reinforcement for other polymers [2,3,4]. Moreover, with outstanding characteristics, including biocompatibility, non-toxicity, high porosity, and good liquid-holding capacity, BC has been applied in various biomedical applications [5], such as wound dressings [6], drug delivery [7], and scaffolds for tissue engineering [8].

To tailor BC for specific applications and broaden its utility, further modification of its properties is necessary. In the context of tissue engineering, the limited biodegradability of BC in the human body poses a significant restriction [9]. To address this challenge, both in situ and ex situ functionalization methods are commonly used. One of the most extensive functionalization techniques is periodate oxidation. Strong ions (IO_4_^−^) oxidize the C2–C3 bond of the glucopyranoside ring and form two aldehyde groups located at the C2 and C3 positions per glucose unit [10], named dialdehyde bacterial cellulose (DBC). The dialdehyde functional group can crosslink polymers consisting of the amine functional group through the Schiff’s base reaction. For example, keratin was crosslinked by DBC to enhance its mechanical strength for use as an adsorbent in the removal of dye and heavy metal ions [11]. To obtain an antibacterial and healing wound dressing without toxicity, chitosan was self-crosslinked with dialdehyde carboxymethyl BC instead of using glutaraldehyde or formaldehyde as a crosslinker [12].

In addition, the aldehyde functional groups modified on the cellulose polymer chain can develop BC characteristics, including water solubility, biodegradability, and antibacterial activity. Li et al. (2009) [9] prepared DBC with an aldehyde content of 60.3% and tested its biodegradation in vitro. DBC could degrade in DI water and phosphate-buffered saline, resulting in mass losses of approximately 50% and 80%, respectively, in 60 days. Furthermore, approximately 10% of the aldehyde content of DBC showed a 16% degradation in Tris-HCl-buffered synthetic body fluid over a 14-day period [13]. Moreover, cellophane was oxidized with sodium periodate by varying reaction times (2–6 h) to develop antimicrobial activity [14]. Aldehyde content or the degree of oxidation (DO) directly influences DBC properties. Most publications reported only a specific value of aldehyde content.

The DO via periodate oxidation is influenced by four independent factors: periodate concentration (the mole ratio of BC to periodate), temperature, reaction time, and pH. Achieving the desired characteristics of DBC regarding DO is quite challenging. Furthermore, minimizing reaction time and the costs for chemicals and utilities is essential. The effects of each parameter must be thoroughly examined, as the study of their impacts and interactions remains insufficient. In this recent work, BC was oxidized using NaIO_4_ to produce DBC with varying DO levels. The Design of Experiment (DOE) with Central Composite Design (CCD) approach-based Response Surface Methodology (RSM) was employed to analyze the impact of three independent factors: mole ratio between BC and NaIO_4_, temperature, and reaction time on DO and DBC properties. A quadratic equation analyzed through RSM was applied to predict the reaction conditions for generating DBC with specific DO levels of 20%, 40%, 60%, 80%, and 100%. The chemical and physical characteristics of DBCs were analyzed. To give an idea, DBCs with specific DO levels were used as reinforcement and crosslinkers for gelatin sponge fabrication. The chemical and physical characteristics of the gelatin–DBC (GDB) sponge, affected by DO levels, were examined to provide insights for further applications.

## 2. Materials and Methods

### 2.1. Bacterial Cellulose (BC)

BC cubes (1 × 1 × 1 cm^3^) synthesized by *Acetobacter xylinum* were kindly supported by Fruitia Food Processing Co., Ltd., Nakorn Pathom, Thailand. The BC was initially treated with 1% NaOH and then rinsed with DI water to achieve a neutral pH. Next, to prepare the BC slurry, the BC cubes were homogenized using a blender (HR2096, Philips, Drachten, The Netherlands) at 1200 rpm for 3 min [15] and stored at 4 °C until use. The dry weight of the BC slurry was controlled at 1 g dry BC/100 g slurry.

### 2.2. Chemicals

Sodium periodate (NaIO_4_) and hydroxylamine hydrochloride were purchased from Alfa-Aesar, Ward Hill, MA, USA. Gelatin Type A (300 bloom), 2,4,6-trinitrobenzenesulfonic acid (TNBS), and 7.5% sodium bicarbonate were supplied by Sigma-Aldrich, St. Louis, MO, USA.

### 2.3. Synthesis of Dialdehyde Bacterial Cellulose (DBC)

BC slurry was mixed with NaIO_4_ in mole ratios of 1:1, 1:1.5, and 1:2. The mixture was stirred at 250 rpm in the dark at 40, 50, and 60 °C for 4, 8, and 12 h. Afterward, the DBC was obtained and rinsed with deionized (DI) water to remove excess NaIO_4_ and NaIO_3_ (byproduct). The DBC slurry was stored at 4 °C until use.

### 2.4. Experimental Design

Factorial design has been extensively applied to experimental design to study the impact of each independent variable on others. Many experiments must be conducted, wasting time and resources. Design-Expert software (Version 7.0, Stat-Ease Inc., Minneapolis, MN, USA) was used in this study to minimize the number of experimental runs. CCD was utilized to design a range of independent parameters, including the mole ratio of BC to NaOI_4_ ($X_{1}$), temperature ($X_{2}$), and time ($X_{3}$). The total number of experimental runs designed was 19, comprising six axial points, eight factorial points, and five central points. The central point was repeated five times to ensure accuracy. The parameters for the central points included a mole ratio of 1:1.5, a temperature of 50 °C, and a reaction time of 8 h. Statistical analysis was performed using Analysis of Variance (ANOVA). RSM was applied to examine the interaction between parameters through three-dimensional (3D) surface plots and a quadratic equation (Equation (1)).

(1) $$Y=\beta_{0}+\sum_{i=1}^{n}\beta_{i}x_{i}+\sum_{i=1}^{n}\beta_{ii}x_{i}^{2}+\sum_{i=1}\sum_{j=i+1}^{n}\beta_{ij}x_{i}x_{j}$$

where Y is a DO of DBC; $\beta_{0}$ is a constant; $\beta_{i}$, $\beta_{ii}$, and $\beta_{ij}$ are linear, quadratic, and second-order interaction coefficients, respectively; $x_{i}$ and $x_{j}$ are independent variables; and n is the number of variables.

### 2.5. Fabrication of Gelatin–DBC (GDB) Sponge

A 15 wt.% gelatin solution was prepared at 60 °C and then mixed with DBC slurry in a 1:1 weight ratio while controlling the DBC concentration at 1% weight (dry basis). The mixture was stirred at 60 °C for 30 min. Afterwards, 50 g of the mixture was poured into a mold with a surface area of 6.5 × 15 cm^2^. Finally, it was lyophilized at −40 °C until fully dried. The composite sponge was stored in a desiccator at room temperature until use.

### 2.6. Characterization of DBC and GDB Sponge

Five replicates were performed for all investigations, excluding FT-IR and SEM.

#### 2.6.1. Fourier Transform-Infrared Spectrometer (FT-IR)

A freeze-dried sample was crushed into a fine powder and scanned using an FT-IR spectrometer (PerkinElmer, Spectrum One, Waltham, MA, USA) between 4000 and 400 cm^−1^ with a resolution of 4 cm^−1^.

#### 2.6.2. Scanning Electron Microscopy (SEM)

Morphologies of BC, DBCs, and GBD sponges were examined by SEM (JEOL, JSM-IT500HR, Tokyo, Japan). The BC and DBC slurries were freeze-dried before coating with a thin layer of gold. For the GBD sponges, they were directly coated with gold. Then, all the samples were observed at a voltage of 5 kV under high resolution.

#### 2.6.3. Determination of Degree of Oxidation (DO)

Adapted from published work [16], 0.1 g dry weight (m) of a sample was mixed with 30 mL DI water and adjusted to pH 4 using 1 M NaOH or HCl. The mixture was added with 20 mL of 0.25 M hydroxylamine hydrochloride solution and then shaken at 150 rpm. After 24 h, the supernatant from the mixture was titrated with 0.1 M NaOH ($M_{NaOH}$). A volume of 0.1 M NaOH used for the titration of the sample ($V_{s}$) was recorded and compared with that used for titration BC ($V_{b}$), which was used as a blank. The molecular weight of BC is 160 g/mol. The DO was calculated by Equation (2).

(2) $$DO\;\left(\%\right)=160\times 0.1\times M_{NaOH}\times (V_{s}-V_{b})/m$$

#### 2.6.4. Degree of Crosslinking

According to Thongsrikhem et al. (2022) [15], the quantitative percentage of occupied amino acid compared to the total amino acid of gelatin was examined using 2,4,6-trinitrobenzenesulfonic acid (TNBS) as a reagent. The aliquot obtained from the reaction was measured for optical density at 346 nm using a UV–visible spectrophotometer (PG Instruments T80+, Leicestershire, UK). Equation (3) was used to calculate the degree of crosslinking. A represents the optical density, and W denotes the weights of the sample (s) and the control (c), which is native gelatin sponge.

(3) $$Degree\;of\;crosslinking\;\left(\%\right)=1-((A_{s}/W_{s})/(A_{c}/W_{c}))\times 100$$

#### 2.6.5. Swelling and Weight Loss

The GDB sponge was cut into 1 × 1 cm^2^ and weighed ($W_{i}$) before being immersed in distilled water. After 24 h, the immersed sponge had its excess water removed by paper wipe and reweighed ($W_{w}$) for calculating swelling ability using Equation (4). The same procedure was carried out to determine weight loss, but the immersion duration was extended to 7 days. Then, the sponge was removed from the water and dried at 60 °C for 24 h. The dried sponge was weighed again ($W_{f}$). The percentage weight loss was calculated using Equation (5).

(4) $$Swelling\;\left(times\right)=(W_{w}-W_{i})/W_{i}$$

(5) $$Weight\;loss\;\left(\%\right)=100\times (W_{i}-W_{f})/W_{i}$$

#### 2.6.6. Porosity

The porosity of the sponge was determined using the liquid replacement technique [17]. A 1 × 1 cm^2^ sponge was measured for dimensions (width × length × thickness = $V_{t}$), weighed ($W_{i}$), then immersed in 99.7% hexane (J.T. Baker, Phillipsburg, NJ, USA) under vacuum conditions, allowing for the complete penetration of hexane into the sponge’s interior without causing any swelling or shrinkage. The sponge was reweighed ($W_{h}$) after reaching equilibrium. The porosity of the sponge was calculated using Equation (6).

(6) $$Porosity\;\left(\%\right)=100\times (W_{h}-W_{i})/(hexane\;density\times V_{t})$$

#### 2.6.7. Mechanical Test

Following ASTM D882, sponge (1 × 10 cm^2^) strength was analyzed by a Universal Testing Machine (Instron model 1123, Norwood, MA, USA) with a tensile rate of 2 mm/min. Tensile strength and elongation at break were obtained.

### 2.7. Statistical Analysis

Regression analysis and Analysis of Variance (ANOVA) were used to analyze the experimental data by considering a significance level (α) of 0.05. The coefficient of determination (R^2^), adjusted coefficient of determination (R^2^_adj_), and predicted coefficient of determination (R^2^_pred_) were employed to validate the obtained quadratic model. The optimization conditions were considered using the desirability function.

## 3. Results and Discussion

DBC was synthesized through periodate oxidation using NaIO_4_ to oxidize the hydroxyl groups to create aldehyde groups within the BC structure. The experimental conditions were designed by DOE, and the simulated equation was employed to predict the conditions needed to achieve the specific DO of DBC. Model validation was conducted to ensure the accuracy of the obtained equation. After producing the DBC with varying levels of DO, it was incorporated into gelatin, serving as both a crosslinker and a reinforcement for fabricating the GDB sponges. The sponges were characterized based on their chemical and physical properties to reveal the influence of the DO level of DBC.

### 3.1. Experimental Design and Data Analysis

The experimental design consisted of 19 experiments, as summarized in Table 1, along with predicted and actual DO (Y) values and residuals (the difference between the actual and predicted DO). A second-order polynomial equation (Equation (7)) describes the relationship between DO and the process parameters. A high degree of agreement between the predicted and actual experimental DO is demonstrated in residual terms (Table 1). Most residuals were quite low, indicating the success of the correlation development between DO and the reaction parameters. According to the actual DO values of runs 8 to 12 (the central point), it was confirmed that the DBC synthesis was highly reproducible. Moreover, the experimental conditions, designed using a full factorial design (27 experimental runs), were also carried out. Results of DO and DBC yield were summarized in the Supplementary File (Table S1).

(7) $$\begin{array}{c} Y=-134.69-17.41X_{1}+5.05X_{2}+2.21X_{3}+0.82X_{1}X_{2}+2.04X_{1}X_{3}\\ -0.007X_{2}X_{3}-3.52X_{1}^{2}-0.05X_{2}^{2}-0.08X_{3}^{2} \end{array}$$

ANOVA was used to assess the reliability of the quadratic model, the impact of each individual factor, and the combined effect of two factors that significantly influenced the experimental results. The data analysis from ANOVA is presented in Table 2 and in Table S2 in the Supplementary Materials (a full version). The regression model showed statistical significance with a high f-value (53.46) and a very low probability (<0.0001). Additionally, it was determined that $X_{1}$, $X_{2}$, and $X_{3}$ were highly significant terms in the model. However, $X_{1}X_{2}$ and $X_{1}X_{3}$ become significant terms when the probability was considered at <0.05. Nagpal et al. (2019) [18] suggested that the value of “Prob less than f″ < 0.05 indicated significant model terms.

Lack of fit was significant in this model, resulting in a 0.02% chance. The f-value of the lack of fit was this large due to noise. The R^2^ of 0.9816 implied that 98.16% of the DO of DBC was affected by the variation in the independent variables. Based on general principles, the difference between R^2^_adj_ and R^2^_pred_ was less than 0.2, implying reasonable agreement between the two. Adeq precision represents the ratio between signal and noise.

**Figure 1:**
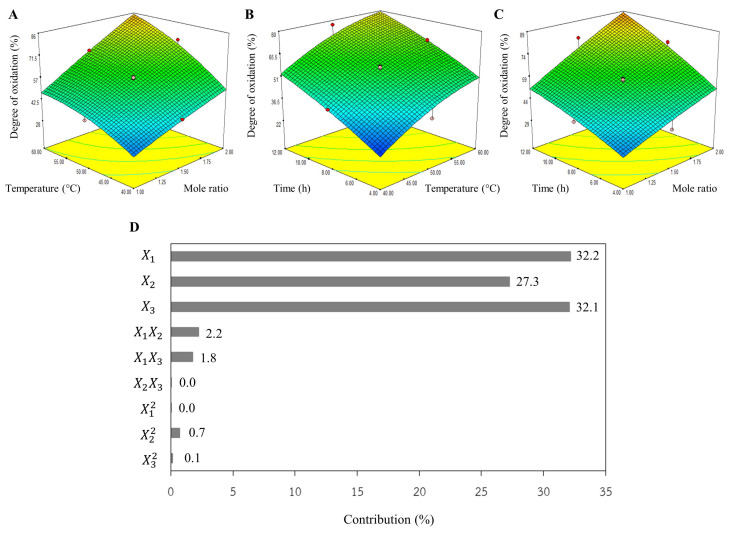
The 3D plots of aldehyde content for (**A**) temperature vs. mole ratio, (**B**) time vs. mole ratio, (**C**) time vs. temperature, and (**D**) percent contribution of each parameter.

From the 3D plot (Figure 1), the DO increased as the mole ratio, temperature, and reaction time rose. It had been reported that an increase in sodium periodate concentration enhances the number of periodate ions, leading to the rapid oxidation of secondary hydroxyl groups located on the carbon atoms in positions 2 and 3 in the structure of cellulose bacteria [16,19]. Considering Figure 1C, the shape of the 3D plot resembles a rhombus without curves. This suggests that the mole ratio and reaction time significantly influence DO, exhibiting a linear relationship. This aligns well with Figure 1D, which demonstrates a strong contribution from the mole ratio (X_1_) and reaction time (X_3_), at 32.2% and 32.1%, respectively. However, the impact of both mole ratio and temperature (X_1_X_2_) was slightly higher than that of both mole ratio and time (X_1_X_3_). There were no impacts from either temperature and time (X_2_X_3_) or squared mole ratio (X_1_^2^). This finding was also observed when the reaction temperature was controlled at 35°C; the reaction time needed to be prolonged to 26 h to obtain dialdehyde carboxymethyl cellulose with 78.6% DO [20].

Consequently, these three parameters all significantly influenced the DO level. Economically, operating costs can be dramatically reduced by minimizing NaIO_4_ utilization, which can be accomplished through either increasing temperature or extending reaction duration. Conversely, high productivity is one of the most critical parameters for industry; therefore, the reaction time should be minimized through the utilization of a high molar ratio and elevated operational temperature. The simulated model could effectively assist in selecting operating conditions based on the investment cost.

Next, DBCs with specific DOs (20%, 40%, 60%, 80%, and 100%) were synthesized, designated as DBC20, DBC40, DBC60, DBC80, and DBC100, respectively. The second-order polynomial equation (Equation (7)) was used to predict the conditions, as shown in Table 3. The DBC was quantitatively analyzed for the DO level to validate the obtained model. The results revealed that the quadratic model had high precision, with an error value (a percentage of difference between DO_actual_ and DO_required_) of ≤5.3%. Therefore, this model had great potential for predicting the reaction conditions to achieve the specific DO of DBC. The DBC was further analyzed for its characteristics.

### 3.2. Characterization of DBC

#### 3.2.1. FT-IR Analysis

**Figure 2:**
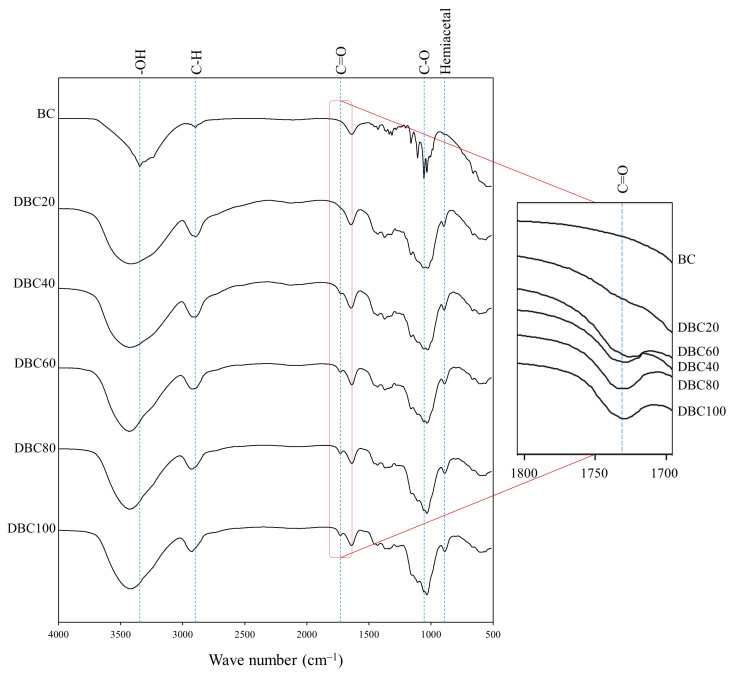
FT-IR spectrum of DBC with different DO levels.

FT-IR spectra of BC and DBC are displayed in Figure 2. The characteristic bands of BC were observed at 3341 cm^−1^ for OH stretching, 2896 cm^−1^ range for C-H, and 1107 cm^−1^ for C-O stretching [21]. All characteristic peaks of BC were also noticed in all DBCs.

New peaks at 1729 cm^−1^ and 886 cm^−1^ in DBC were observed, representing the aldehyde group (C=O) and the formation of hemiacetal bonds between aldehyde groups and neighboring hydroxyl groups, respectively. Wegrzynowska-Drzymalska et al. (2022) [22] also observed the aldehyde peak and hemiacetal bond peaks of dialdehyde cellulose at 1740 cm^−1^ and 880 cm^−1^, respectively. In the zoomed-in figure at 1729 cm^−1^, the intensities tended to be broadened with an increase in DO. It confirmed that the hydroxyl groups located at C2 and C3 of the D-glucose structure were oxidized by IO_4_^−^ to form aldehyde groups, and this was in accordance with the DO level.

#### 3.2.2. Morphologies of BC and DBCs

**Figure 3:**
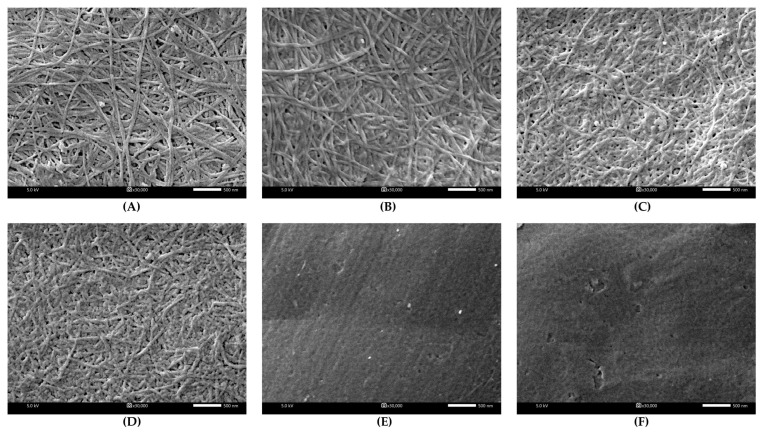
Morphologies of BC (**A**), DBC20 (**B**), DBC40 (**C**), DBC60 (**D**), DBC80 (**E**), and DBC100 (**F**).

The structure of BC and DBCs is illustrated in Figure 3. A fibrous web-like network structure was observed in BC (Figure 3A). BC fibers were approximately 52.3 ± 6.6 nm in diameter (determined using ImageJ software, v1.54m). A similar morphology of BC was also reported in [23]. After BC underwent oxidation by periodate, ultrafine fibers, thinner than their original sizes, were observed in the following order: DBC20 (43.2 ± 6.0 nm), DBC40 (34.7 ± 6.5 nm), and DBC60 (27.3 ± 5.7 nm). It is also acknowledged in the synthesis of dialdehyde microcrystalline cellulose that the fiber length became shorter than its original length from 56.2 to 33.6 μm [24]. However, the fibrous structure could not be seen in 80DBC and 100DBC due to the harsh conditions: high mole ratio, high temperature, and prolonged reaction time, which strongly oxidized hydroxyl groups at C2 and C3 in glucose monomers, resulting in the weakening of the BC polymeric backbone and degradation of the fibrous structure. Tachai et al. (2024) [20] also found that periodate oxidation transformed a smooth surface of carboxymethyl cellulose into a pitted and non-smooth surface.

The influence of various characteristics of DBC on the physical and mechanical properties of the gelatin crosslinked with DBC (GDB) sponge was further investigated.

### 3.3. Characterization of GDB Sponge

Gelatin, a protein biopolymer, is derived from the partial hydrolysis of collagen, exhibiting remarkable biocompatibility comparable to that of collagen, with more cost-effectiveness [25]. Due to the unique characteristics of gelatin, including its gel-forming ability, biodegradability, and promotion of cell attachment, proliferation, and differentiation [26], gelatin has been widely utilized in biomedical [27,28], tissue engineering [29,30], and pharmaceutical [31] applications. Gelatin is composed of various types of amino acids linked together by peptide bonds, with three primary amino acids: glycine (25–29%), proline (15–17%), and hydroxyproline (12–14%) [32]. Due to the high water solubility of gelatin, many applications require structural stability in the wet state; therefore, gelatin must be crosslinked through a Schiff’s base reaction with substances containing aldehyde groups, such as glutaraldehyde and formaldehyde [33]. Nonetheless, these two chemicals are toxic to both animal and human cells, rendering them unsuitable for use in biomedical applications [34,35]. An alternative option is natural extracts such as cinnamaldehyde [15] and genipin [36], which are relatively expensive. Seeking a crosslinker with non-toxicity and biocompatibility is a challenging task.

Low mechanical strength is the weakest point of gelatin, which limits its applications in several areas [37]. Since neat gelatin is brittle and fragile, reinforcement with other polymers is necessary. For example, cellulose nanocrystals incorporated with electrospun gelatin nanofibers to improve their tensile strength for further utilization in biomedical fields [38]. Chitosan–gelatin hydrogel reinforced with cellulose nanofibrils showed enhancements in mechanical strength and thermal stability for diabetic wound healing applications [39]. Moreover, gelatin-starch was successfully integrated with polyvinyl alcohol to modify mechanical resilience [40]. The high potential of utilizing gelatin must include crosslinking and reinforcement steps. Consequently, the recent work proposed DBC simultaneously acting as a crosslinker and a reinforcing agent to improve the structural stability of gelatin sponge in both dry and wet states by controlling the DO level of DBC.

#### 3.3.1. Degree of Crosslinking of GDB Sponge

DBC served simultaneously as a crosslinker and a reinforcement for the fabrication of gelatin sponges. The degree of crosslinking between the amino groups of gelatin and the aldehyde groups in DBC is summarized in Table 4. The results reveal that the degree of crosslinking increased with higher aldehyde content/DO. Due to the existing aldehyde content in DBC, it actively interacts with the amine functional groups of gelatin through Schiff’s base reaction [41]. For the lowest aldehyde content (GDB20), the degree of crosslinking was only 12.6%. In contrast, the maximum degree of crosslinking achieved was nearly 50% using DBC100. However, the degree of crosslinking of the gelatin film crosslinked with dialdehyde cellulose was reported to be about 68–75% [41]. This might be attributed to the 9% lower gelatin concentration compared to recent work.

FT-IR spectroscopy was used to characterize the sponges (Figure S1 in the Supplementary Materials). The characteristic bands of gelatin and DBC were present in all the GDB sponges. The vibration of the OH bond in gelatin and DBC peaks between 3100 and 3600 cm^−1^ was observed. After gelatin was crosslinked with DBC, the OH peaks shifted from 3284 cm^−1^ to 3279–3273 cm^−1^. This shift could be attributed to the hydrogen bonding interaction between gelatin and DBC. Tohamy (2025) [42] also observed a shift in the OH peak from a higher (3336 cm^−1^) to a lower (3330 cm^−1^) wavenumber, indicating strong hydrogen bonding between dialdehyde cellulose and chitosan. This finding was also reported in the dialdehyde carboxylated cellulose crosslinked with soy protein [43]. For the chemical reaction (Schiff’s base), a new peak of imine (C=N) around 1600–1700 cm^−1^ or a shift in the 1650–1600 cm^−1^ range [44] should be detected. However, this peak overlapped with the amide I of gelatin and the carbonyl peaks of DBC, leading to unclear identification. Moreover, the characteristic peaks (1729 and 886 cm^−1^) of DBC disappeared in all GDB sponges, which was also reported in [41], indicating that these two peaks were utilized by the crosslinking process.

#### 3.3.2. Weight Loss of GDB Sponge

The sponges were immersed in water for 7 days to determine the structural stability in terms of weight loss percentage, which directly relates to the degree of crosslinking. Generally, gelatin must be crosslinked to enhance its water resistance before being utilized in many applications. The percent weight loss of the GDB is presented in Table 4. Without crosslinking, the GL sponge had the highest weight loss compared to the GDB sponges. After crosslinking, the structure of the sponge was more stable in water, retaining more than 85% of its weight (GDB40, GDB60, GDB80, and GDB100) after 7 days. Due to the strong chemical reaction (Schiff’s base reaction) between gelatin and DBC, DBC possessed the potential to be a crosslinker for gelatin. Consequently, DBC containing 40% DO could significantly improve the structural stability of gelatin.

#### 3.3.3. Swelling of GDB Sponge

The swelling ability of the composite depends on several parameters, such as hydrophilicity/hydrophobicity, material structure, and interaction between polymer chains. Although gelatin and DBC both exhibit high hydrophilicity, after crosslinking, the swelling decreased as the DO of the DBC increased. Particularly with GDB80 and GDB100, the sponges could only swell to 4.7 times their original state. This may be due to the high aldehyde content in DBC effectively bonding with the gelatin polymer chains, resulting in a more rigid structure, which corresponds with the degree of crosslinking and percent weight loss. This finding aligns with gelatin gel crosslinked with dialdehyde cellulose nanocrystal [45] and dialdehyde starch [46].

#### 3.3.4. Morphology of GDB Sponge

**Figure 4:**
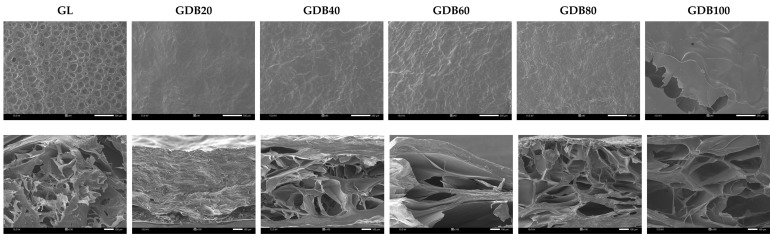
SEM images of the surface area with a magnification of ×40 (**above**) and cross-sectional area with a magnification of ×100 (**below**) of the GL and GDB sponges.

The fabrication technique employed in this research is lyophilization, which creates a three-dimensional porous structure suitable for various applications, including scaffolds for tissue engineering [47], wound dressings [48], and wastewater treatment [49]. After ice crystals are formed during the freezing step, they are sublimated during the drying phase, resulting in a porous structure, particularly in the interior of the sponge [17]. The thickness and porosity of the sponges are shown in Table 4, where the GL sponge exhibited the highest thickness. In contrast, the GDB20 structure, when gelatin crosslinked with DBC20, resulted in a densely non-porous film-like structure, as depicted in Figure 4 (second row). Due to the mild conditions, the structure of DBC20 (Figure 3B) retained fibers, similar to BC, without being destroyed by oxidation reactions. When gelatin was crosslinked with the DBC20 fibers, they tended to compact together, resulting in a film-like structure with a thickness of 0.2 mm. As the aldehyde content of DBC increased, the thickness and porosity of the GDB sponges reached their maximum at 60% DO. As seen in Figure 3, a fibrous network structure was observed in DBC20, DBC40, and DBC60, demonstrating a gradual trend of fiber degradation. With increasing DO, the fiber diameter and structure became thinner and looser, respectively. All these parameters substantially influenced the GDB sponge structure.

For the GDB80 sponge, the thickness and porosity decreased when compared to those of the GDB60. According to the oxidation conditions (Table 3), a doubled reaction time was operated for synthesizing DBC80 with nearly the same mole ratio and temperature, resulting in a high degree of degradation in the fibrous structure. The harsh conditions could damage the BC fibers. When comparing GDB80 and GDB100, greater thickness and porosity were observed in the gelatin crosslinked with DBC100. This suggests that DBC100 was well-incorporated into gelatin, resulting in a homogeneous composite sponge, which was obviously noticed on the smooth surface of GDB100 (Figure 4, first row).

The surface area of the sponges is shown in Figure 4 (1st row). A high roughness with numerous pits was noticed on the surface of the native gelatin (GL) sponge. This appearance was also observed in [17]. When gelatin was crosslinked with 20DBC (GDB20), all the pits disappeared and the surface became much smoother, implying a good distribution of 20DBC fibers within the gelatin. However, the GDB40 and GDB60 sponges exhibited greater surface roughness compared to the GDB20 sponge. This may be due to the smaller diameter of the fibers creating more roughness on the surface. For the non-fibrous structure of DBC80, the roughness on the surface of the GDB80 sponge was fine. In contrast, a homogeneous and smooth surface resembling an open-area sponge was only observed on the surface of the GDB100 sponge due to the finest and non-fibrous structure of DBC100.

#### 3.3.5. Mechanical Strength of GDB Sponge

The tensile strength and elongation at break of the GL and GDB sponges are gathered in Table 4. Gelatin is recognized as a biopolymer with high strength but low elasticity. As shown in Table 4, the GL sponge exhibited quite high strength with the lowest elongation at break. Crosslinking with DBC, considering different DO and structure, enhanced the tensile strength of the GDB sponges as the DO of the DBC increased. The high degree of crosslinking contributed to the improved mechanical strength of the sponges. The structural integrity could be improved by the chemical reaction between amine and aldehyde groups, resulting in greater tensile strength [41]. Only the homogeneous sponge (GDB100) achieved the highest tensile strength of 9 MPa, representing a 52.5% improvement over that of the GL sponge.

After the gelatin sponges were crosslinked with DBC, the elongation at break showed huge improvement. This can be attributed to both the crosslinking and reinforcing processes between gelatin and DBC. This finding aligns with the results reported by Lin et al. (2019) [50]. The elasticity of all gelatin sponges crosslinked with DBCs improved by approximately 20–26% compared to the native GL sponge. An insignificant difference was observed in all GDB sponges. Generally, the enhancement of tensile strength conversely correlates with improved elasticity. Surprisingly, DBC could simultaneously enhance tensile strength and elongation at break.

### 3.4. Future Applications for DBC

DBC demonstrated significant potential as both a crosslinker and a reinforcing agent for protein biopolymers. DBC nanofiber was used to crosslink and reinforce the gelatin/carboxymethyl chitosan hydrogel for three-dimensional (3D) printing tissue engineering applications [51]. The 3D sponge-shaped collagen scaffolds were crosslinked with dialdehyde cellulose to enhance mechanical strength, and their ability to promote embryonic nerve cell culture was also demonstrated, confirming high biocompatibility [52]. To fabricate a rehydratable hydrogel for wound healing, quaternized chitosan was crosslinked with DBC to enhance its mechanical properties [53]. Not only are biomedical fields applied, but gelatin film was also crosslinked with dialdehyde cellulose to develop tensile strength, water resistance, and barrier properties for application in the packaging area [41].

For biological properties, the 6.59 mmol/g (100%) aldehyde content present in the dialdehyde microcrystalline cellulose (DAMC) structure exhibited a strong scavenging effect on 1,1-diphenyl-2-picrylhydrazyl (DPPH), (2,2′-Azino-bis-(3-ethyl-benzthiazoline-6-sylfonic acid (ABTS), and hydroxyl radicals, with half-inhibitory concentration (IC_50_) values of 5.9, 5.6, and 8.1 mg/mL, respectively, indicating high antioxidant activity [24]. Along with its antibacterial activity, DAMC showed minimum inhibitory concentrations (MICs) of 15 mg/mL against *Staphylococcus aureus*, *Bacillus subtilis*, and *Escherichia coli,* respectively, and 30 mg/mL against *Salmonella typhimurium*. Gram-negative bacteria had greater sensitivity to DAMC compared to Gram-positive bacteria [54]. Moreover, DBC was biocompatible and promoted fibroblast attachment and proliferation on day 4 [55].

In conclusion, cellulose oxidized by periodate exhibited distinctive characteristics, including biocompatibility, biodegradability, and antioxidant and antibacterial activities, which may potentially be applied in various fields as a crosslinking and reinforcing agent to improve the mechanical properties and structural stability of protein-type biopolymers.

## 4. Conclusions

The preparation of DBC using a 1:2 molar ratio at 60 °C for 12 h yielded the highest aldehyde content of 98.3%. The molar ratio of BC and NaIO_4_, as well as the reaction time, had a slightly greater effect on aldehyde content formation than temperature. The simulated quadratic model was highly accurate, with an error of only 5.3%. DO level significantly influenced the chemical and morphological structure of DBC. Gelatin was crosslinked and reinforced with DBCs containing DO/aldehyde content levels of 20%, 40%, 60%, 80%, and 100%. The GDB80 and GDB100 sponges exhibited excellent structural stability in water due to their high degree of crosslinking. The morphology of the sponge was influenced by both DO and the structure of the DBC. The strongest sponge was GDB100, featuring the highest tensile strength and elongation at break. The distinct characteristics of the GDB sponge will make it suitable for various applications, depending on the requirements, which can be adjusted by the DBC’s DO.

## Figures and Tables

**Table 1:** Degree of oxidation (DO) from prediction and experiment and their residuals.

Run	Conditions			DO		
	Mole Ratio of BC and NaIO_4_	Temperature	Time	Predicted	Actual	Residual
	$\left(X_{1}\right)$	$\left(X_{2}\right)$	$\left(X_{3}\right)$			
1	1:1 (−1)	40 (−1)	4 (−1)	15.6	18.3	2.7
2	1:1 (−1)	40 (−1)	12 (+1)	37.7	36.3	−1.4
3	1:1 (−1)	50 (0)	8 (0)	42.1	40	−2.1
4	1:1 (−1)	60 (+1)	4 (−1)	34.7	36.4	1.7
5	1:1 (−1)	60 (+1)	12 (+1)	55.8	55	−0.8
6	1:1.5 (0)	40 (−1)	8 (0)	39.3	39.1	−0.2
7	1:1.5 (0)	50 (0)	4 (−1)	41.5	35.2	−6.3
8	1:1.5 (0)	50 (0)	8 (0)	57.6	57.7	0.1
9	1:1.5 (0)	50 (0)	8 (0)	57.6	57.6	0
10	1:1.5 (0)	50 (0)	8 (0)	57.6	56.7	−0.9
11	1:1.5 (0)	50 (0)	8 (0)	57.6	57.2	−0.4
12	1:1.5 (0)	50 (0)	8 (0)	57.6	57.8	0.2
13	1:1.5 (0)	50 (0)	12 (+1)	71.2	78	7.6
14	1:1.5 (0)	60 (+1)	8 (0)	66.1	66.8	−0.7
15	1:2 (+1)	40 (−1)	4 (−1)	28.5	29.2	0.7
16	1:2 (+1)	40 (−1)	12 (+1)	66.9	65.2	−1.7
17	1:2 (+1)	50 (0)	8 (0)	71.3	73.9	2.6
18	1:2 (+1)	60 (+1)	4 (−1)	64	65.3	1.3
19	1:2 (+1)	60 (+1)	12 (+1)	101.4	98.6	−2.8

**Table 2:** ANOVA data analysis for the model.

Source	Model	$X_{1}$		$X_{2}$	$X_{3}$	$X_{1}X_{2}$		$X_{1}X_{3}$	$X_{2}X_{3}$	$X_{1}^{2}$		$X_{2}^{2}$	$X_{3}^{2}$	Lack of Fit Data
Sum of Squares	6598.22	2137.44		1795.60	2211.17	133.66		133.66	0.5512	2.12		65.11	4.14	122.60
df	9	1		1	1	1		1	1	1		1	1	5
Mean Square	733.14	2137.44		1795.60	2211.17	133.66		133.66	0.5512	2.12		65.11	4.14	24.52
f-value	53.46	155.87		130.94	161.24	9.75		9.75	0.04	0.15		4.75	0.30	119.61
*p*-value	<0.0001	<0.0001		<0.0001	<0.0001	0.0110		0.0198	0.8120	0.7434		0.1045	0.4477	0.0002
R^2^			0.9816				Adeq precision				31.9295			
R^2^_adj_			0.9633				C.V. (%)				6.87			
R^2^_pred_			0.8537											

**Table 3:** Predicted conditions for synthesizing the specified DO of DBC.

Symbol	Predicted Conditions			DO (%)		% Error
	Mole Ratio of BC and NaIO_4_ (Time)	Temperature (°C)	Time (h)	Required	Actual	
DBC20	1:1.29	40	4	20	20.8 ± 0.1	4.0
DBC40	1:1.45	50	4	40	42.1 ± 0.2	5.3
DBC60	1:1.82	60	4	60	62.4 ± 0.5	4.0
DBC80	1:1.86	60	8	80	82.8 ± 0.9	3.5
DBC100	1:1.97	60	12	100	98.0 ± 0.6	2.0

% Error = |(DO_actual_ − DO_required_)/DO_required_) × 100|.

**Table 4:** Characteristics of gelatin (GL) and GDB sponges.

Sponge	Thickness (mm)	Porosity (%)	Degree of Crosslinking (%)	Weight Loss After 7 Days (%)	Swelling After 1 Day (Time)	Tensile Strength (MPa)	Elongation at Break (%)
GL	4.0 ± 0.4	23.9 ± 3.1	0.0 ± 0.0	52.3 ± 5.4	15.3 ± 2.9	5.9 ± 1.0	29.1 ± 5.6
GDB20	0.2 ± 0.0	3.5 ± 3.1	12.6 ± 2.8	33.0 ± 2.5	11.8 ± 0.6	2.8 ± 1.6	51.7 ± 8.8
GDB40	1.7 ± 0.3	32.3 ± 9.8	19.9 ± 2.7	15.0 ± 0.8	7.5 ± 0.8	4.6 ± 4.3	49.3 ± 5.0
GDB60	3.2 ± 0.6	41.3 ± 5.6	35.6 ± 2.3	11.4 ± 0.6	5.9 ± 1.3	4.9 ± 1.2	50.1 ± 6.8
GDB80	2.3 ± 0.4	28.3 ± 9.5	45.6 ± 1.6	10.6 ± 0.8	4.7 ± 0.2	6.8 ± 4.4	50.4 ± 8.6
GDB100	2.8 ± 0.2	47.7 ± 2.3	49.6 ± 1.6	9.6 ± 0.7	4.7 ± 0.2	9.0 ± 3.6	55.0 ± 2.6

## Data Availability

The original contribution presented in this study is included in the article. Further inquiries can be directed to the corresponding author.

## Supplementary Materials

The following supporting information can be downloaded at: https://www.mdpi.com/article/10.3390/polym17131836/s1, Figure S1: FT-IR spectrum of GL and GDB sponges.; Table S1: ANOVA analysis for the response function Y.; Table S2: Degree of oxidation (DO) and yield of full factorial experimental design.
