# Supplementary material for: Tailoring Dialdehyde Bacterial Cellulose Synthesis for Versatile Applications
Source: Polymers (Basel). 2025 Jun 30;17(13):1836. doi: 10.3390/polym17131836 (PMC12251641; doi:10.3390/polym17131836)
Supplement: Supplementary file 1 [file polymers-17-01836-s001.zip › polymers-3702664-supplementary.pdf]

Article

Supplementary Materials: Tailoring Dialdehyde Bacterial Cellulose Synthesis for Versatile Applications

Krittanan Kadsanit [1], Malinee Sriariyanun [2,3], Muenduen Phisalaphong [4] and Suchata Kirdponpattara [1,3,*]

[1] Department of Chemical Engineering, Faculty of Engineering, King Mongkut's University of Technology North Bangkok, Bangkok 10800, Thailand; krittanankadsanit@gmail.com
[2] The Sirindhorn International Thai-German Graduate School of Engineering, King Mongkut's University of Technology North Bangkok, Bangkok 10800, Thailand; malinee.s@tggs.kmutnb.ac.th
[3] Biorefinery and Process Automation Engineering Center (BPAEC), King Mongkut's University of Technology North Bangkok, Bangkok 10800, Thailand
[4] Department of Chemical Engineering, Faculty of Engineering, Chulalongkorn University, Bangkok 10330, Thailand; muenduen.p@chula.ac.th
* Correspondence: suchata.k@eng.kmutnb.ac.th; Tel.: +66-2-555-200

Table S1. ANOVA analysis for the response function Y.

Source	Sum of Squares	df	Mean Square	f-value	p-value
Model	6598.22	9	733.14	53.46	< 0.0001
X_1	2137.44	1	2137.44	155.87	< 0.0001
X_2	1795.60	1	1795.60	130.94	< 0.0001
X_3	2211.17	1	2211.17	161.24	< 0.0001
X_1X_2	133.66	1	133.66	9.75	0.0123
X_1X_3	133.66	1	133.66	9.75	0.0123
X_2X_3	0.5512	1	0.5512	0.0402	0.8456
X_1^2	2.12	1	2.12	0.1548	0.7031
X_2^2	65.11	1	65.11	4.75	0.0573
X_3^2	4.14	1	4.14	0.3022	0.5959
Residual	123.42	9	13.71		
Lack of fit	122.60	5	24.52	119.61	0.0002
Pure error	0.8200	4	0.2050		
Cor total	6721.64	18			

Std. Dev. = 3.70, Mean = 53.91, C.V. = 6.78%.

R^2 = 0.9816, R^2_{adj} = 0.9633, R^2_{pred} = 0.8537, Adeq precision = 31.9295.

Table S2. Degree of oxidation (DO) and yield of full factorial experimental design.

Run	Conditions			DO (%)	DBC yield (g DBC/g BC)
	Mole ratio of BC and $NaIO_4$ (time)	Temperature (°C)	Time (h)		
1	1:1	40	4	18.3 ± 0.5	0.78 ± 0.02
2	1:1	40	8	27.5 ± 0.1	0.82 ± 0.04
3	1:1	40	12	36.3 ± 0.4	0.73 ± 0.07
4	1:1	50	4	24.9 ± 1.3	0.80 ± 0.01
5	1:1	50	8	40.0 ± 0.6	0.77 ± 0.02
6	1:1	50	12	53.0 ± 0.1	0.67 ± 0.03
7	1:1	60	4	36.4 ± 0.4	0.73 ± 0.01
8	1:1	60	8	52.4 ± 1.8	0.64 ± 0.01
9	1:1	60	12	55.0 ± 0.8	0.48 ± 0.02
10	1:1.5	40	4	22.4 ± 0.1	0.80 ± 0.01
11	1:1.5	40	8	39.1 ± 1.2	0.79 ± 0.01
12	1:1.5	40	12	50.6 ± 0.3	0.82 ± 0.06
13	1:1.5	50	4	35.2 ± 1.0	0.77 ± 0.501
14	1:1.5	50	8	57.7 ± 0.1	0.68 ± 0.01
15	1:1.5	50	12	78.0 ± 1.3	0.66 ± 0.01
16	1:1.5	60	4	52.9 ± 1.0	0.66 ± 0.01
17	1:1.5	60	8	66.8 ± 0.2	0.51 ± 0.04
18	1:1.5	60	12	79.4 ± 0.1	0.39 ± 0.02
19	1:2	40	4	29.2 ± 0.7	0.81 ± 0.01
20	1:2	40	8	51.7 ± 0.4	0.76 ± 0.01
21	1:2	40	12	65.2 ± 0.4	0.82 ± 0.04
22	1:2	50	4	45.5 ± 1.6	0.71 ± 0.04
23	1:2	50	8	73.9 ± 0.3	0.64 ± 0.02
24	1:2	50	12	88.8 ± 0.9	0.61 ± 0.04
25	1:2	60	4	65.3 ± 2.3	0.58 ± 0.01
26	1:2	60	8	80.5 ± 0.7	0.37 ± 0.02
27	1:2	60	12	98.6 ± 0.5	0.23 ± 0.01

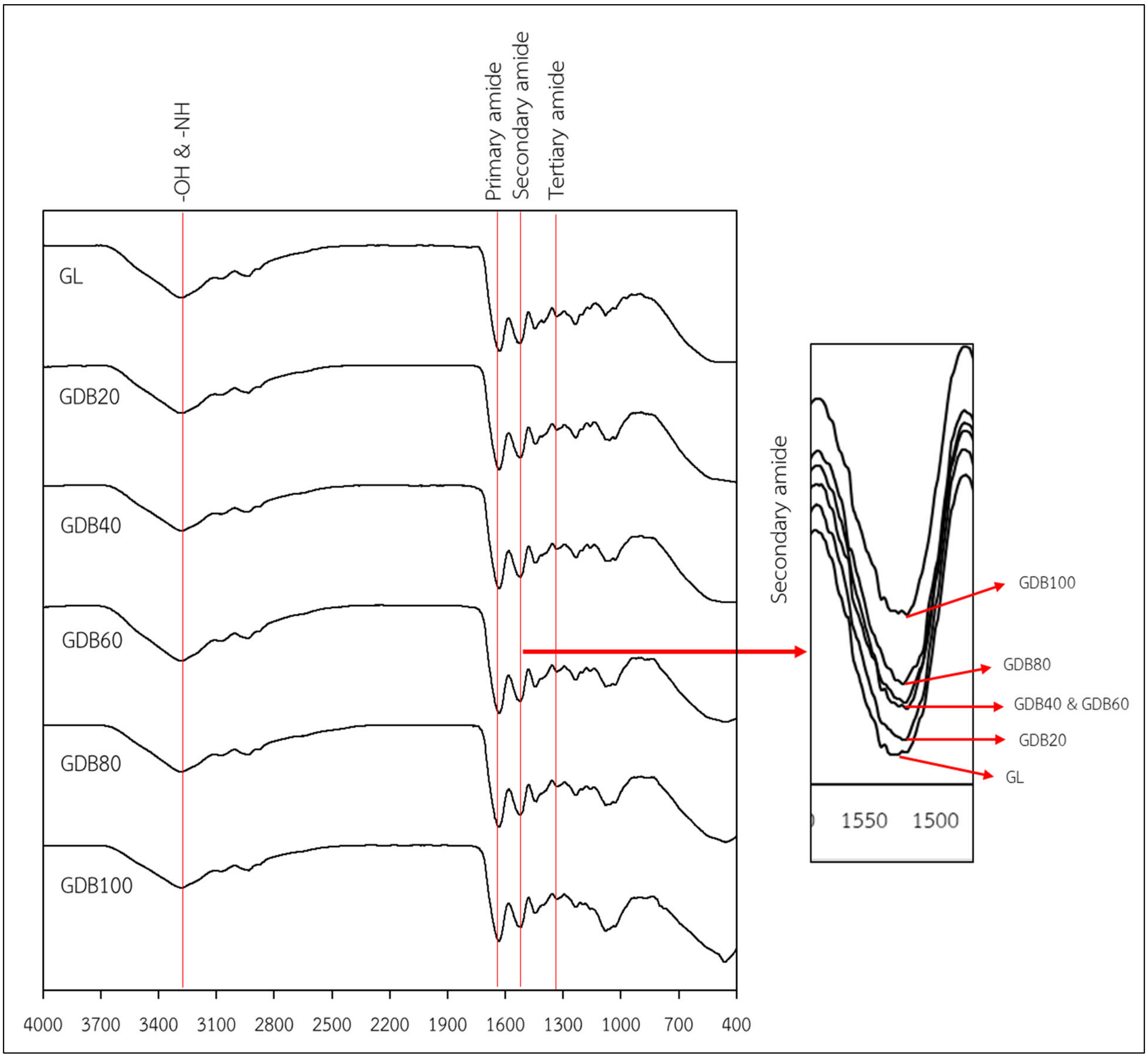

Figure S1. FT-IR spectrum of GL and GDB sponges.
